# Atypical Presentation of Pilar Cyst Over the Volar Aspect of the Thumb: A Case Report and Review of Literature

**DOI:** 10.7759/cureus.55296

**Published:** 2024-02-29

**Authors:** Suneel Ramanujapuram, Apurve Parameswaran, Murtuza A Mohammed, Anil K Reddy, Krishna K Eachempati

**Affiliations:** 1 Department of Orthopaedics and Hand Surgery, Medicover Hospitals, Hyderabad, IND

**Keywords:** pilar cyst, trichilemmal cyst, thumb lesion, epithelial cyst, benign sebaceous cyst

## Abstract

Pilar cysts are derived from the outer layer of the root sheath of hair follicles. They were conventionally thought to arise from hair-bearing skin like the scalp. However, this notion has been refuted recently. Pilar cysts of the hand are extremely rare, with only a few case reports in the literature. We report the case of a 40-year-old male patient, with no known medical co-morbidities, who presented with a swelling over his left thumb. It was occasionally painful, and caused difficulty in grasping objects. Physical examination revealed a 2.5 x 1.5 cm swelling over the volar aspect of the thumb, at the level of the proximal phalanx. MRI revealed the presence of a well-defined cystic lesion superficial to the flexor tendons. The possibility of an epidermal cyst was considered, and the patient was advised surgery in view of his symptoms and progression in the size of the swelling. He underwent excision of the lesion along with a segment of adherent skin. Histopathological examination of the lesion revealed the presence of a pilar cyst. The patient did not have recurrence of symptoms following surgery, and was found to be doing well at the three-year follow-up. This case report urges a re-thinking of the possible origins of pilar cysts from atypical locations.

## Introduction

Pilar cysts (also known as trichilemmal cysts) were first identified by Pinkus [1]. They are derived from the outer layer of the root sheath, from the deeper part of hair follicles, and comprise a keratinized epidermal wall of stratified squamous epithelium without a granular layer, surrounding semi-solid hair keratin [2]. They appear as smooth, firm white-walled cysts without a punctum, and can easily be enucleated [2]. They commonly arise on the scalp where hair follicles are abundant, but can appear, in rare cases, in the axilla, groin, face, trunk, and extremities [3]. Though conventionally thought to arise only from hair-bearing skin, this notion has been disproved recently [3,4].

Pilar cysts of the hand are extremely rare, with only six case reports in the literature, to the authors’ knowledge [3-8]. Five of these reported cysts arising from the dorsal surface or at the level of the distal phalanx of the digits, with possible origins from hair follicles or the nail matrix [3-7], while one reported a cyst over the volar aspect of the index finger with a history of prior trauma [8]. We present a case of pilar cyst over the volar aspect of the thumb at the level of the proximal phalanx, with no history of trauma.

## Case presentation

A 40-year-old male patient, a teacher by profession, with no known medical co-morbidities, presented at our outpatient department with complaints of a swelling over his left thumb. He first noticed the swelling one year prior to presentation. It gradually increased in size, eventually causing difficulty in grasping objects with his hand. It was occasionally painful. There was no history of trauma, fever, discharge, or other similar swellings elsewhere over the body. Family history was not significant.

**Figure 1:**
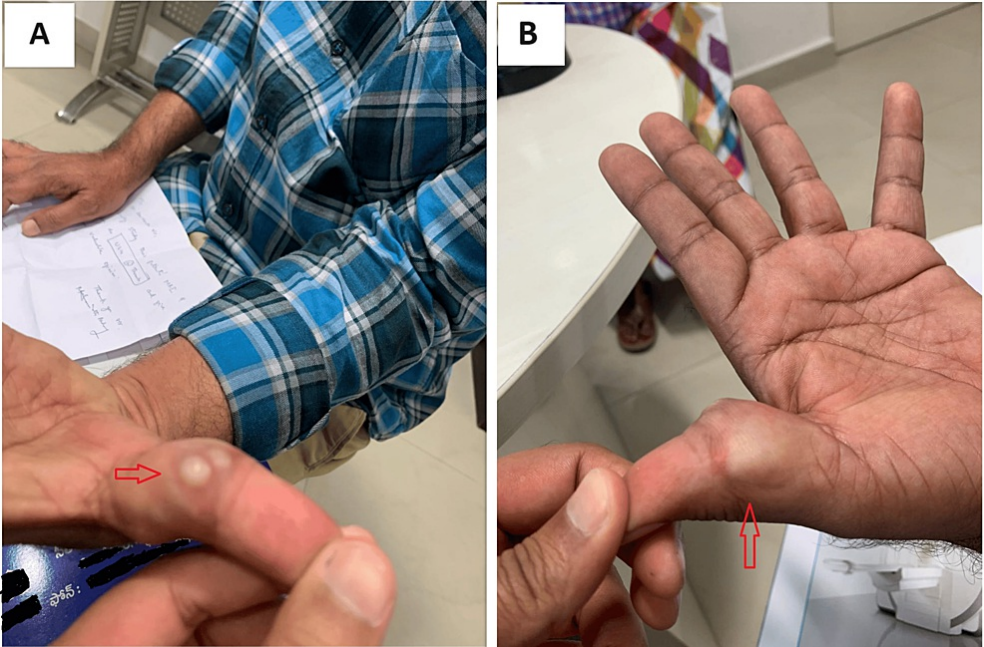
Clinical appearance of the swelling The swelling was noted over the volar aspect of the thumb at the level of the proximal phalanx, and spanned the mediolateral extent of the thumb. (A) shows the ulnar aspect of the thumb while (B) shows the radial aspect of the thumb; the red arrows point to the lesion.

On examination, a 2.5 x 1.5 cm swelling was noted over the volar aspect of the left thumb, spanning the mediolateral extent of the thumb at the level of the proximal phalanx (Figure 1). It was globular in shape, with a smooth surface and soft consistency. The margins of the swelling were discrete. The skin over the swelling was not pinchable, but the swelling was not adherent to the underlying bone or muscles. The distal aspect of the thumb appeared well-perfused. Movements of the thumb were not restricted. No sensory or motor deficits were elicited. 

**Figure 2:**
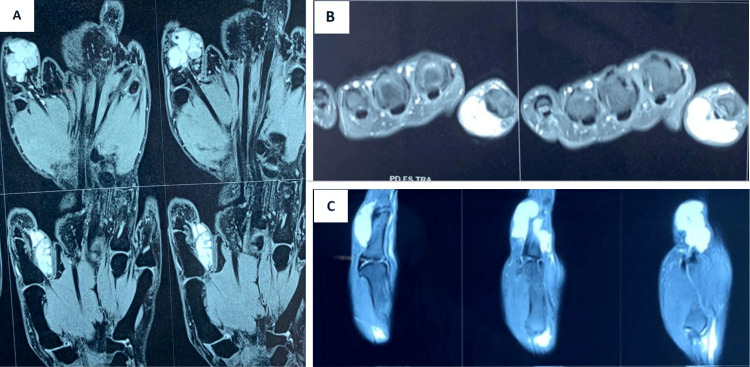
MRI images of the lesion A well-defined lobulated septate lesion is noted in the subcutaneous plane over the volar aspect of the proximal phalanx of the thumb, superficial to the flexor tendon. (A) shows the coronal sections, (B) shows axial sections, and (C) shows sagittal sections of the thumb.

MRI of the thumb was advised. It revealed the presence of a well-defined, lobulated cystic lesion with thin septa in the subcutaneous plane, on the ventral surface of the thumb, superficial to the flexor tendons (Figure 2). The underlying bone appeared normal. The possibility of an epidermal cyst was considered. In view of occasional pain, difficulty holding objects, and progression in the size of the swelling, the patient was advised surgical excision of the lesion.

**Figure 3:**
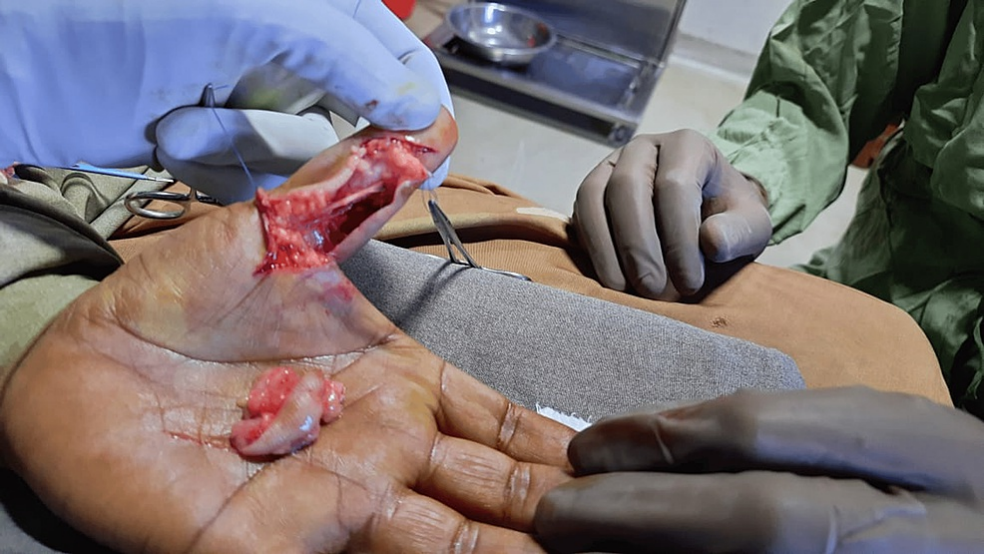
Intra-operative image following excision of the lesion The lesion was excised along with a segment of adherent skin and sub-dermal tissue; the underlying flexor pollicis longus tendon was found to be intact.

Under brachial block, using a volar Bruner incision, the lesion was exposed. A well-defined, greyish-brown swelling, adherent only to the sub-dermal tissue was noted. It was found overlying the tendon sheath of the flexor pollicis longus, encircling the digital neurovascular bundles. It was surrounded by loose areolar tissue and was easily dissected from the surrounding tissues. It was excised with a segment of skin (Figure 3). The wound was closed with 4-0 polypropylene sutures.

**Figure 4:**
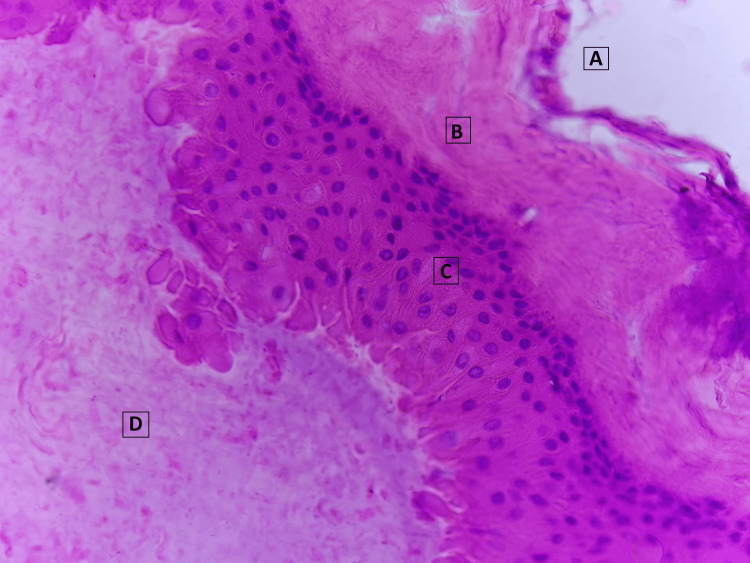
Histopathologic appearance of the cyst (A) represents the exterior of the cyst, while (B) represents the surrounding fibrous tissue. The cyst lining was composed of stratified squamous epithelium without a granular layer (C). The lumen contained keratinous material (D).

The resected lesion was sent for a biopsy. Macroscopic examination showed a brownish-grey soft mass, measuring 2 x 1 x 0.6 cm. Histopathological examination revealed the presence of a cyst lined by stratified squamous epithelium without a granular layer, which is characteristic of pilar cysts (Figure 4). No nuclear atypia was noted. The lumen of the cyst was filled with compact, wet keratinous debris. These findings were suggestive of a pilar cyst. The patient did not have recurrence of symptoms and was found to be doing well, with painless thumb movement and no difficulty in gripping objects, during the three-year postoperative follow-up visit.

## Discussion

Epithelial cysts comprise epidermal cysts (80%) and pilar cysts (20%) [3]. Pilar cysts cannot be distinguished from epidermal cysts clinically and require histopathological examination [3]. They are noted in 5-10% of the general population, with a strong female preponderance [9]. Around 90% pilar cysts occur in the scalp region where hair follicles are abundant [3]. They are usually solitary, but could occasionally present as multi-focal lesions [4,10-11]. The previously held notion that they could not arise from non-hair-bearing skin like in the palms or soles, has been challenged, and found to be untrue [4].

Four reports in the literature present cases of patients with pilar cysts arising from non-hair-bearing skin at the level of the distal phalanx of fingers [4-7]. It has been proposed that these cysts probably originated from the nail matrix, since its keratinization is trichilemmal [4]. Kim et al. reported a case of a laryngeal pilar cyst [9]. Ikegami et al. reported a case of pilar cyst over the pulp of the index finger, with a prior history of trauma [8]. The current case is unique, since it presents the findings and management of a pilar cyst over the volar aspect of the thumb at the level of the proximal phalanx, with no antecedent history of trauma, which could not have originated from a hair follicle or the nail matrix.

Pilar cysts are generally simple to treat since they can easily be excised [2]. However, they could be complicated by additional pathology. Rupture of the capsule of the cyst, or imbalances between matrix metalloproteinases and their tissue inhibitors, could result in inflammation of the cyst, which would warrant delayed surgical excision [3,12]. Cysts may be infected with the human papillomavirus [13]. Calcification of the cyst, producing radio-opacity on radiographs, has also been reported [5,6].

On rare occasions, pilar cysts might progress to “proliferating pilar tumors” (PTT), which could undergo malignant transformation, and require radiation therapy and/or chemotherapy in addition to surgical excision [3,5,14]. PTTs are aggressive, invasive lesions, and could result in considerable morbidity or even mortality [14]. Clinicians managing patients with a suspected pilar cyst must always consider the possibility of malignant transformation. Multi-focal pilar cysts with the potential for malignant transformation have also been reported in the literature, which present challenges in diagnosis and management [11]. Genetic mechanisms for the pathogenesis of pilar cysts have been explored, and might provide insights regarding hereditary pilar cysts and their pathophysiology [2,15].

The patient in the current report had a benign pilar cyst with keratinous material inside the lumen, in an unusual location, with no history of prior trauma. The possibility of this lesion was not considered in the differential diagnoses prior to surgery, and the histopathological findings were unforeseen. Following surgical excision of the lesion, the patient’s symptoms resolved immediately. In the three years following surgery, he had no complaints and was found to be doing well at his latest follow-up visit.

## Conclusions

This case report re-emphasizes the possibility of pilar cysts originating from atypical and non-hair-bearing skin. It also demonstrates the need to counsel patients with seemingly benign cystic lesions, when managed conservatively, regarding the possibility of progression of the swelling requiring a re-evaluation of management options. Additionally, it illustrates the importance of histopathological examination of surgically excised cystic lesions of uncertain etiology. Lastly, it urges a re-thinking of the possible origins of pilar cysts.

## References

[1] Pinkus H (1969). "Sebaceous cysts" are trichilemmal cysts. Arch Dermatol.

[2] Eiberg H, Hansen L, Hansen C, Mohr J, Teglbjaerg PS, Kjaer KW (2005). Mapping of hereditary trichilemmal cyst (TRICY1) to chromosome 3p24-p21.2 and exclusion of beta-CATENIN and MLH1. Am J Med Genet A.

[3] Liu M, Han H, Zheng Y, Xiao S, Feng Y (2020). Pilar cyst on the dorsum of hand: a case report and review of literature. Medicine (Baltimore).

[4] Maheshwari K, Hindocha S, Yousif A (2019). Rare presentation of pilar cyst of the thumb. World J Plast Surg.

[5] Melikoglu C, Eren F, Keklik B, Aslan C, Sutcu M, Zeynep Tarini E (2014). Trichilemmal cyst of the third fingertip: a case report. Hand Surg.

[6] Kwon KE, Kim SJ, Kim JH (2018). Imaging sonographic findings of in a case of proliferating trichilemmal tumor of a finger: a case report. J Clin Ultrasound.

[7] El Hassani Y, Beaulieu JY, Tschanz E, Marcheix PS (2013). Proliferating trichilemmal tumor of the pulp of a finger: case report and review of the literature [Article in French]. Chir Main.

[8] Ikegami T, Kameyama M, Orikasa H, Yamazaki K (2003). Trichilemmal cyst in the pulp of the index finger: a case report. Hand Surg.

[9] Kim CM, Holliday MA, Newkirk KA (2018). Laryngeal pilar cyst masquerading as an internal/external laryngocele. Clin Med Insights Ear Nose Throat.

[10] Ramaswamy AS, Manjunatha HK, Sunilkumar B, Arunkumar SP (2013). Morphological spectrum of pilar cysts. N Am J Med Sci.

[11] Asilian A, Siadat AH, Shahmoradi Z, Shariat S, Moghadam NA, Soozangar H (2016). Multiple giant pilar cyst distributed over the body since childhood. Indian J Dermatol.

[12] Velez AM, Brown VM, Howard MS (2011). An inflamed trichilemmal (pilar) cyst: not so simple?. N Am J Med Sci.

[13] Nanes BA, Laknezhad S, Chamseddin B, Doorbar J, Mir A, Hosler GA, Wang RC (2020). Verrucous pilar cysts infected with beta human papillomavirus. J Cutan Pathol.

[14] Kim UG, Kook DB, Kim TH, Kim CH (2017). Trichilemmal carcinoma from proliferating trichilemmal cyst on the posterior neck. Arch Craniofac Surg.

[15] Hörer S, Marrakchi S, Radner FP (2019). A monoallelic two-hit mechanism in PLCD1 explains the genetic pathogenesis of hereditary trichilemmal cyst formation. J Invest Dermatol.

